# Inhibition of PI3K/mTOR increased the sensitivity of hepatocellular carcinoma cells to cisplatin via interference with mitochondrial‐lysosomal crosstalk

**DOI:** 10.1111/cpr.12609

**Published:** 2019-04-29

**Authors:** Jiyao Sheng, Luyan Shen, Liankun Sun, Xuewen Zhang, Ranji Cui, Lizhong Wang

**Affiliations:** ^1^ Department of Hepatobiliary and Pancreatic Surgery The Second Hospital of Jilin University Changchun Jilin China; ^2^ Department of Pathophysiology, College of Basic Medical Sciences Jilin University Changchun Jilin China; ^3^ Jilin Provincial Key Laboratory on Molecular and Chemical Genetic The Second Hospital of Jilin University Changchun Jilin China

**Keywords:** chemotherapy resistance, hepatocellular carcinoma, lysosomal biogenesis, mitochondrial‐lysosomal crosstalk, mitophagy

## Abstract

**Objectives:**

The genotoxicity of cisplatin towards nuclear DNA is not sufficient to explain the cisplatin resistance of hepatocellular carcinoma (HCC) cells; cisplatin interacts with many organelles, which can influence the sensitivity. Here, we explored the role of mitochondrial‐lysosomal crosstalk in the cisplatin resistance of HCC cells.

**Materials and methods:**

Huh7 and HepG2 cells were subjected to different treatments. Flow cytometry was conducted to detect mitochondrial reactive oxygen species, mitochondrial mass, lysosomal function, mitochondrial membrane potential and apoptosis. Western blotting was performed to evaluate protein levels. The oxygen consumption rate was measured to evaluate mitochondrial function.

**Results:**

Cisplatin activated mitophagy and lysosomal biogenesis, resulting in crosstalk between mitochondria and lysosomes and cisplatin resistance in HCC cells. Furthermore, a combination of cisplatin with the phosphatidylinositol‐3‐kinase/mammalian target of rapamycin (PI3K/mTOR) inhibitor PKI‐402 induced lysosomal membrane permeabilization. This effect changed the role of the lysosome from a protective one to that of a cell death promoter, completely destroying the mitochondrial‐lysosomal crosstalk and significantly enhancing the sensitivity of HCC cells to cisplatin.

**Conclusions:**

This is the first evidence of the importance of mitochondrial‐lysosomal crosstalk in the cisplatin resistance of HCC cells and of the destruction of this crosstalk by a PI3K/mTOR inhibitor to increase the sensitivity of HCC cells to cisplatin. This mechanism could be developed as a novel target for treatment of HCC in the future.

## 1 INTRODUCTION

Cisplatin (cis‐diamminedichloroplatinum(II), CDDP), as a representative platinum drug, has shown efficacy in hepatocellular carcinoma (HCC) treatment,^1^, ^2^, ^3^, ^4^ but most hepatobiliary cancer guidelines do not recommend cisplatin as a first‐line treatment because of the low sensitivity of this drug to HCC.^5^, ^6^, ^7^, ^8^, ^9^ Cisplatin has also been shown to induce DNA damage; however, recent studies have found that genotoxicity accounts for only a small portion of the cytotoxicity of this drug.^10^ Cisplatin can also interact with mitochondria, lysosomes, the endoplasmic reticulum and other organelles,^11^, ^12^ influencing the sensitivity of tumour cells to cisplatin. Therefore, identification of the target of cisplatin in HCC cells is very important for elucidation of the resistance mechanism.

Several studies, including our own, have reported that cisplatin enhanced the reactive oxygen species (ROS) levels in HCC cells.^13^, ^14^, ^15^ This finding provides support for mitochondria as targets of cisplatin in HCC because these organelles are the major sites of ROS formation in the cell,^16^, ^17^ and the production of mitochondrial ROS (mtROS) is an important indicator of damaged mitochondrial antioxidant defence function.^18^ Furthermore, low mtROS levels can activate the mitophagy‐lysosome pathway to degrade damaged mitochondria and mtROS.^19^, ^20^, ^21^, ^22^ This phenomenon plays a protective role in cells^23^ but has not been reported in the context of HCC chemotherapy. However, high levels of mtROS accumulation were seen to be closely associated with mitochondrial apoptosis in tumour cells.^24^, ^25^, ^26^

mtROS are also important for the maintenance of lysosomal homeostasis. To maintain autophagic flux, tumour cells require the quantity and function of lysosomes to be maintained via lysosomal biogenesis and autophagic lysosomal reformation.^27^ Low levels of ROS can activate transcription factor EB (TFEB)‐mediated lysosomal biogenesis, ensuring mitophagy,^28^ while high levels of ROS contribute to lysosomal membrane permeabilization (LMP), leading to the lysosomal release of cathepsin and hydrolase into the cytoplasm, causing apoptosis.^29^, ^30^, ^31^ Therefore, we speculate that the mtROS levels in HCC cells play a key role in the maintenance of the homeostasis of mitochondria and lysosomes in the mitophagy‐lysosome pathway.

The highly activated phosphatidylinositol‐3‐kinase (PI3K)/mammalian target of rapamycin (mTOR) pathway in HCC cells is an important connector pathway involved in mitochondrial‐lysosomal crosstalk. This pathway is involved in the regulation of mitochondrial metabolism and in the resistance to mitochondrial pathway apoptosis.^32^ Kirstein found that the PI3K inhibitor BKM120 impaired mitochondrial function,^33^ while inhibition of PI3K/mTOR increased the ROS levels in tumour cells.^34^, ^35^, ^36^, ^37^ Additionally, Madge reported that PI3K signalling was involved in the control of lysosomal activity and stability,^38^ and Seitz showed that the dual PI3K/mTOR inhibitor NVP‐BEZ235 stimulated enlargement of the lysosomal compartment, generated ROS and cooperated with chloroquine (CQ) to trigger LMP in neuroblastoma cells.^39^ However, it remains unclear whether chemotherapeutic drugs combined with PI3K/mTOR inhibitors cause LMP.

In this study, we found that mtROS induced the mitophagy‐lysosome pathway and lysosome biogenesis in response to cisplatin in HCC cells. We also demonstrated that mitochondrial‐lysosomal crosstalk was involved in the resistance to cisplatin‐induced apoptosis in HCC cells and in the maintenance of the cells in a stable state. When cisplatin was combined with the PI3K/mTOR inhibitor PKI‐402, the mtROS levels in HCC cells increased significantly, thereby destroying the stable state. High mtROS levels mediated LMP, resulting in mitochondrial injury, which produced additional mtROS and aggravated mitochondrial and lysosomal damage, thus forming a vicious cycle and eventually leading to HCC cell apoptosis. This study provides novel insights into potential comprehensive treatments of HCC.

## 2 MATERIALS AND METHODS

### 2.1 Materials and reagents

MTT was purchased from Sigma‐Aldrich (USA). Chloroquine diphosphate, E‐64, PKI‐402 and rapamycin were purchased from MedChemExpress (USA). The primary antibodies against LC3B, SQSTM1/p62, TFEB, PINK1, parkin, Fis1, Drp1, Mfn1 and Mfn2 were purchased from Abcam (USA), and LAMP1, beta‐actin, alpha‐tubulin, cathepsin B, cathepsin D and cytochrome c were purchased from Proteintech (USA).

### 2.2 Cell culture

The human HCC cell lines Huh7 and HepG2 were purchased from the Chinese Academy of Medical Sciences (China). The two cell lines were incubated at 37°C in 5% CO_2_ and cultured in DMEM supplemented with 10% foetal bovine serum (FBS), 100 IU/mL penicillin and 100 µg/mL streptomycin.

### 2.3 Cell metabolic activity assays

Cells were seeded in 96‐well plates. After exposure of the cells to different concentrations of cisplatin, 500 μg/mL MTT solution was added, and the cells were maintained for 4 hours at 37°C. Then, DMSO was added. The optical density was measured at 570 nm using a CLARIOstar microplate reader (BMG Labtech GmbH, Germany). The cell metabolic activity was calculated as follows: cell metabolic activity = absorbance of the experimental group/absorbance of the control group × 100%.

### 2.4 Flow cytometric analysis

mtROS levels, mitochondrial mass, mitochondrial membrane potential and apoptosis were assessed by flow cytometric analysis with the stains MitoSOX Red, MitoTracker Green FM (Invitrogen, USA) and JC‐1 and the FITC Annexin V Apoptosis Detection Kit I (BD Pharmingen, USA). In addition, the quantity and activity of the lysosomes were determined by staining with LysoTracker Green DND‐26 and DQ Red BSA (Invitrogen). The samples were examined using a BD Accur C6 Plus personal flow cytometer (BD Biosciences, USA) or a Bio‐Rad S3 cell sorter (Bio‐Rad Laboratories, Inc, USA). Data analysis was performed using FlowJo v10.

### 2.5 Western blotting analysis

Cells subjected to the different desired treatments were harvested and incubated in RIPA for 40 minutes at 4°C to isolate the total protein content. Protein concentrations were analysed using the Bradford Kit (Beyotime Biotechnology, China). For cytosolic protein extraction, we used the Minute Plasma Membrane Protein Isolation and Cell Fractionation Kit (Invent Biotechnologies, Inc, USA). The protein samples were separated by 12% SDS‐PAGE and then transferred to PVDF membranes (Roche, Switzerland). The membranes were blocked with 5% (w/v) skim milk for 2 hours and then incubated with the different desired antibodies at 4°C overnight. Then, the membranes were incubated with the corresponding secondary antibodies at room temperature for 1.5 hours. Immunodetection was performed using ECL reagent (Thermo Fisher Scientific, Inc, USA), and visualization was performed using a Syngene bioimaging system (Synoptics, UK). Protein levels were quantified using Quantity One software and normalized to β‐actin.

### 2.6 Immunofluorescence staining and confocal laser microscopy

Cells were seeded onto coverslips in 24‐well plates overnight and exposed to different experimental conditions, and then, the cells were fixed in 4% (w/v) paraformaldehyde/PBS for 20 minutes. After fixation, the cells were subjected to proteinase K digestion for 1 minute, permeabilized with 0.1% Triton X‐100 for 5 minutes and blocked with bovine serum albumin for 30 minutes. The cells were incubated with primary antibody overnight at 4°C and then stained with FITC‐conjugated secondary antibodies (1:500 dilution) for 30 minutes in the dark. The cells were then treated with Hoechst 33342/H_2_O (1 μg/mL) for 2 minutes. For staining of mitochondria and lysosomes, the cells were incubated with MitoTracker RED (200 nmol/L, 30 minutes), LysoTracker Green (50 nmol/L, 60 minutes) or DQ Red BSA (10 µg/mL, 2 hours). After rinsing with PBS, images were acquired by using an Olympus FV1000 confocal laser microscope (Olympus Corporation, Japan).

### 2.7 Oxygen consumption rate

Cells were plated in 96‐well plates at a density of 8 × 10^4^ cells/well overnight. Then, the medium in all the wells was replaced with a preheated liquid mixture containing reconstituted MitoXpress‐Xtra reagent, fresh culture medium and the different treatments. The wells were then sealed using preheated mineral oil. Fluorescence decay was measured using a CLARIOstar microplate reader.

### 2.8 Reverse transcription‐quantitative polymerase chain reaction analysis

TRIzol (Invitrogen) was used to extract the total cellular RNA, and reverse transcription was performed to generate cDNA, which was amplified using reverse transcription‐quantitative polymerase chain reaction (RT‐qPCR). The primer sequences are listed in Table 1. RT‐qPCR analysis was performed using TransStart Top Green qPCR SuperMix (TransGen Biotech, China) under the following reaction conditions: 94.0°C for 30 seconds, followed by 40 cycles of 94.0°C for 5 seconds and 60.0°C for 30 seconds. Each sample examined in triplicate in a CFX96 Touch real‐time PCR detection system (Bio‐Rad Laboratories, Inc, USA). The mRNA levels were normalized to *Gapdh*. Relative quantification was performed using the $2^{-\Delta \Delta C_{t}}$ method.

**Table 1:** Primer sequences of TFEB and CLEAR network

Gene symbol	Gene name	Forward primer	Reverse primer	Function
*TFEB*	Transcription factor EB	CCAGAAGCGAGAGCTCACAGAT	TGTGATTGTCTTTCTTCTGCCG	Lysosomal biogenesis
*CTSA*	Cathepsin A	CAGGCTTTGGTCTTCTCTCCA	TCACGCATTCCAGGTCTTTG	Lysosomal hydrolase
*CTSB*	Cathepsin B	AGTGGAGAATGGCACACCCTA	AAGAAGCCATTGTCACCCCA	
*CTSD*	Cathepsin D	AACTGCTGGACATCGCTTGCT	CATTCTTCACGTAGGTGCTGGA	
*CTSF*	Cathepsin F	ACAGAGGAGGAGTTCCGCACTA	GCTTGCTTCATCTTGTTGCCA	
*GNS*	Glucosamine (N‐acetyl)‐6‐sulfatase	CCCATTTTGAGAGGTGCCAGT	TGACGTTACGGCCTTCTCCTT	
*TPP1*	Tripeptidyl peptidase I	GATCCCAGCTCTCCTCAATACG	GCCATTTTTGCACCGTGTG	
*MCOLN1*	Mucolipin 1	TTGCTCTCTGCCAGCGGTACTA	GCAGTCAGTAACCACCATCGGA	Lysosomal carrier
*LAMP1*	Lysosomal‐associated membrane protein 1	ACGTTACAGCGTCCAGCTCAT	TCTTTGGAGCTCGCATTGG	Lysosomal‐associated membrane protein
*LAMP2*	Lysosomal‐associated membrane protein 2	GCACAGTGAGCACAAATGAGT	CAGTGGTGTGTATGGTGGGT	
*ATP6VOD2*	ATPase, H+ transporting, lysosomal 38 kDa, V0 subunit D2	TCTCACCTATATGACGTGCAGT	GGTGGCACTTCCCCAGAATTT	Lysosomal acidification
*ATP6V0E1*	ATPase, H+ transporting, lysosomal 9 kDa, V1 subunit E1	CATTGTGATGAGCGTGTTCTGG	AACTCCCCGGTTAGGACCCTTA	
*ATP6V1H*	ATPase, H+ transporting, lysosomal 50/57 kDa, V1 subunit H	GGAAGTGTCAGATGATCCCCA	CCGTTTGCCTCGTGGATAAT	
*CLCN7*	Chloride channel 7	TGATCTCCACGTTCACCCTGA	TCTCCGAGTCAAACCTTCCGA	
*GAPDH*	—	GGAGCGAGATCCCTCCAAAAT	GGCTGTTGTCATACTTCTCATGG	—

### 2.9 RT^2^ profiler PCR array system

The RT^2^ Profiler PCR array system for human mitochondrial energy metabolism (PAHS‐008Z; Qiagen, Germany) profiles the expression of 84 key genes involved in human mitochondrial energy metabolism (Supporting Information Table S1). RT‐qPCRs were performed using a CFX96 Touch Real‐Time PCR detection system. Fluorescence intensities were analysed using Qiagen online software, and relative quantification was performed using the $2^{-\Delta \Delta C_{t}}$ method. Changes in expression of the 84 genes were visualized as a heatmap.

### 2.10 Statistical analysis

All the data are representative of three independent experiments, each performed in triplicate. Statistical significance was analysed using one‐way ANOVA, followed by Tukey or Newman‐Keuls post hoc analysis. The analyses were performed with GraphPad Prism 5.0 statistical software (USA). *P < 0.05 was considered to indicate statistical significance; **P < 0.01 indicated a highly significant difference; and ***P < 0.001 indicated an extremely significant difference.

## 3 RESULTS

### 3.1 Mitochondria are important targets of cisplatin in HCC cells

**Figure 1:**
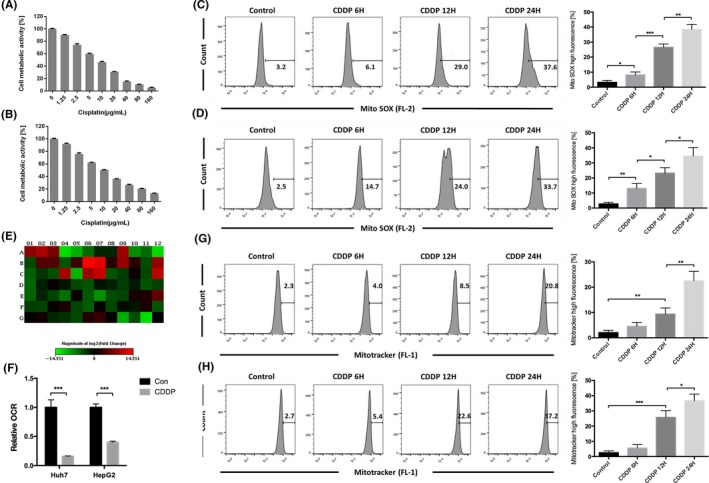
Mitochondria were an important target of cisplatin in HCC cells. A, Huh7 and B, HepG2 cells were treated with varying doses of cisplatin for 24 h. Cell metabolic activity was determined using the MTT assay and expressed as the mean ± SD; n = 3. C, Huh7 cells were treated with 8 μg/mL cisplatin, and D, HepG2 cells were treated with 12 μg/mL cisplatin for varying durations and stained with MitoSOX Red and then detected using flow cytometry. The percentage of cells with high MitoSOX fluorescence is expressed as the mean ± SD; n = 3, *P < 0.05, **P < 0.01, ***P < 0.001. E, Huh7 cells were incubated with 8 μg/mL cisplatin for 8 h and examined using a PCR array. For details regarding gene names, see Supporting Information Table S1. Changes are presented as a heatmap; green indicates downregulation, and red indicates upregulation. Data were derived from three experiments. F, OCR was measured after Huh7 cells were treated with 8 μg/mL cisplatin and HepG2 cells were treated with 12 μg/mL cisplatin. Relative OCR values are expressed as the mean ± SD; n = 3, ***P < 0.001. G, Huh7 cells were treated with 8 μg/mL cisplatin and H, HepG2 cells were treated with 12 μg/mL cisplatin for varying durations. Then, the cells were stained with MitoTracker Green FM and detected using flow cytometry. The percentage of cells with high MitoTracker fluorescence is expressed as the mean ± SD; n = 3, *P < 0.05, **P < 0.01, ***P < 0.001

The MTT assay was used to evaluate cell metabolic activity (Figure 1A,B). MitoSOX Red is a fluorogenic dye for mtROS^40^ that can be used to determine mtROS levels. The mtROS levels increased in HCC cells treated with cisplatin in a time‐dependent manner (Figure 1C,D). Because mtROS accumulation is an important indicator of mitochondrial dysfunction,^41^ mitochondrial energy metabolism was next evaluated by the appropriate PCR array and based on the extracellular oxygen consumption rate (OCR). The PCR array showed that most of the mitochondrial respiratory complex‐related genes were downregulated in Huh7 cells treated with cisplatin (Figure 1E), and cisplatin decreased the OCR in HCC cells (Figure 1F). MitoTracker Green FM staining showed that the fluorescence intensity increased in a time‐dependent manner in HCC cells (Figure 1G,H). These results showed that mitochondria are important targets of cisplatin in HCC cells and that cisplatin can damage mitochondria and increase mtROS levels, leading to accumulation of damaged mitochondria.

### 3.2 Cisplatin induced mitochondrial fission and the mitophagy‐lysosomal pathway in HCC cells

**Figure 2:**
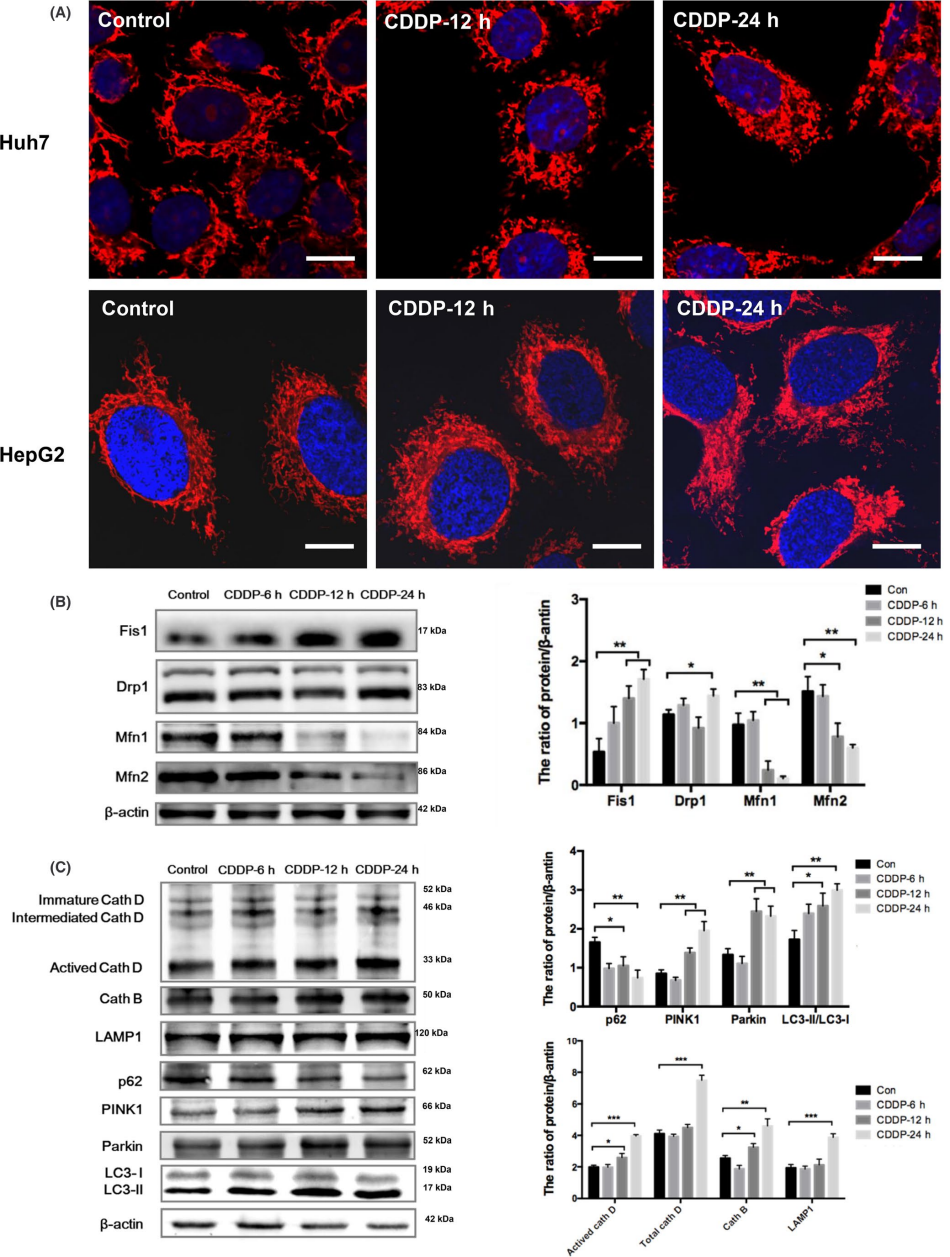
Cisplatin induced mitochondrial fission and the mitophagy‐lysosomal pathway in HCC cells. A, Huh7 cells were treated with 8 μg/mL cisplatin, and HepG2 cells were treated with 12 μg/mL cisplatin for 12 and 24 h. Then, the cells were stained with MitoTracker Red CMXRos and observed by confocal laser microscopy; scale bar = 10 μm. B, Western blot detection of mitochondrial fusion proteins and mitochondrial fission proteins in Huh7 cells treated with 8 μg/mL cisplatin for 6, 12 and 24 h. The protein/beta‐actin ratio is expressed as the mean ± SD; n = 3, *P < 0.05, **P < 0.01. C, Western blot detection of mitophagy‐lysosomal pathway‐related proteins in Huh7 cells treated with 8 μg/mL cisplatin for 6, 12 and 24 h. The protein/beta‐actin ratio is expressed as the mean ± SD; n = 3, *P < 0.05, **P < 0.01, ***P < 0.001

Reactive oxygen species damage mitochondria, which initiates mitochondrial quality control for maintenance of mitochondrial homeostasis.^42^ To determine whether HCC cells overcome cisplatin‐induced mitochondrial damage in this manner, the cells were cultured with cisplatin for various durations, stained with MitoTracker Red CMXRos and observed by confocal microscopy (Figure 2A). In the absence of treatment, the mitochondria in HepG2 and Huh7 cells were long and tubular. After exposure to cisplatin for 12 hours, the mitochondrial morphology was fragmented, exhibiting short tubular shapes. After exposure for 24 hours, the mitochondria appeared as small dots, indicating that cisplatin induced mitochondrial fission in HCC cells.

Selected Huh7 cells with a high degree of mitochondrial fission were examined by Western blotting. After cisplatin treatment, the expression of the mitochondrial fusion proteins Mfn1 and Mfn2 decreased significantly, while that of the mitochondrial fission proteins FIS1 and Drp1 increased significantly (Figure 2B). Mitochondrial fission can isolate damaged mitochondria and degrade these mitochondria via mitophagy.^23^ To further determine whether cisplatin induced mitophagy in HCC cells, we detected the expression of the mitophagy‐lysosome pathway‐related proteins PTEN‐induced putative kinase (PINK)1, parkin, LC3, p62, lysosomal‐associated membrane protein 1 (LAMP1), cathepsin B and cathepsin D in Huh7 cells treated with cisplatin (Figure 2C). After exposure to cisplatin for 12 and 24 hours, the mitophagy‐related proteins PINK1, parkin and LC3‐II/I were upregulated in HCC cells; the mitophagy substrate p62 was downregulated; and the lysosomal surface proteins LAMP1, cathepsin B and cathepsin D were upregulated. These results suggested that cisplatin induced mitochondrial fission in HCC cells and then activated PINK1/parkin‐mediated mitophagy and perhaps lysosome biogenesis.

### 3.3 Cisplatin induced lysosomal biogenesis in HCC cells, contributed to mitophagy and caused synergistic mitochondrial‐lysosomal crosstalk

**Figure 3:**
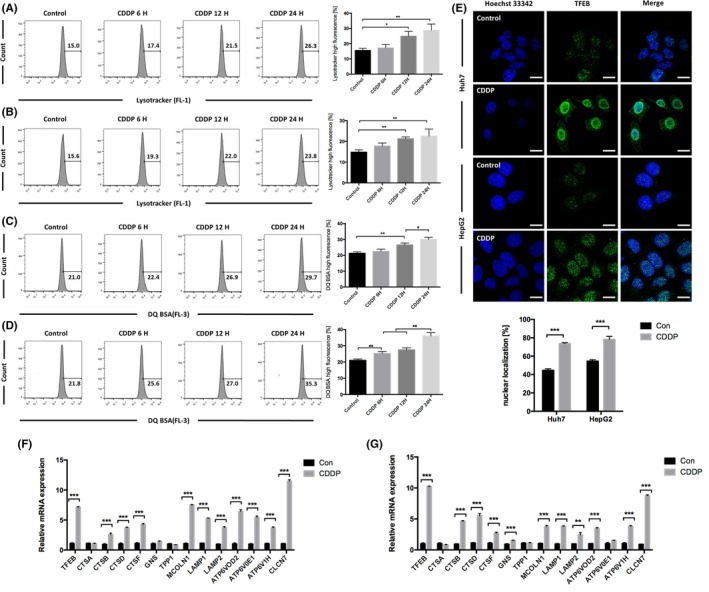
Cisplatin induced lysosomal biogenesis in HCC cells. A, Huh7 cells were treated with 8 μg/mL cisplatin, and B, HepG2 cells were treated with 12 μg/mL cisplatin for varying durations. Then, the cells were stained with LysoTracker Green DND‐26 and detected using flow cytometry. The percentage of cells with high LysoTracker fluorescence is expressed as the mean ± SD; n = 3, *P < 0.05, **P < 0.01. C, Huh7 cells were treated with 8 μg/mL cisplatin, and D, HepG2 cells were treated with 12 μg/mL cisplatin for varying durations. Then, the cells were stained with DQ Red BSA and detected using flow cytometry. The percentage of cells with high DQ Red BSA fluorescence is expressed as the mean ± SD; n = 3, *P < 0.05, **P < 0.01. E, Colocalization of TFEB and nuclei in Huh7 cells treated with 8 μg/mL cisplatin and HepG2 cells treated with 12 μg/mL cisplatin for 8 h; scale bar = 10 μm. The percentage of nuclear localization is analysed by ImageJ and expressed as the mean ± SD; n = 3, ***P < 0.001. F, The mRNA levels of TFEB and the CLEAR system in Huh7 cells treated with 8 μg/mL cisplatin and G, HepG2 cells treated with 12 μg/mL cisplatin for 8 h. Relative mRNA expression is expressed as the mean ± SD; n = 3, **P < 0.01, ***P < 0.001

Mitophagy consumes large numbers of lysosomes.^43^ To verify the enhancement of lysosome biogenesis in HCC cells induced by cisplatin, LysoTracker Green staining was used to quantify lysosome levels. Lysosome numbers were shown to increase in a time‐dependent manner (Figure 3A,B). DQ Red BSA, which is a red BODIPY dye conjugated to bovine serum albumin and the proteolysis of which leads to dequenching and the release of red fluorescence,^44^ was next used to evaluate lysosomal phagocytosis and degradation. The fluorescence intensity of HCC cells treated with cisplatin was observed to increase gradually in a time‐dependent manner (Figure 3C,D). To elucidate the underlying mechanism, we detected the activation of TFEB, a key molecule associated with lysosomal biogenesis. We showed that cisplatin increased the expression of TFEB and activated the translocation of TFEB to the nucleus (Figure 3E). Elements of the coordinated lysosomal expression and regulation (CLEAR) system were previously shown to bind TFEB and initiate lysosomal biogenesis.^45^ Therefore, we examined the expression of key genes in the CLEAR network by RT‐qPCR. We found that cisplatin significantly increased *TFEB* transcription and upregulated the expression of the CLEAR genes including genes of lysosomal membrane proteins *LAMP1*, and *LAMP2*, genes of lysosomal hydrolase *CTSB*,* CTSD* and *CTSF*, genes of lysosomal carrier *MCOLN1*, genes of lysosomal acidification *ATP6V0D2*,* ATP6V1H* and *CLCN7* in HepG2 and Huh7 cells. The expression of lysosomal hydrolase gene *GNS* is upregulated in HepG2 cells treated with cisplatin, and lysosomal acidification gene *ATP6V0E1* is upregulated in Huh7 cells treated with cisplatin. But cisplatin did not increase lysosomal hydrolase gene *CTSA* and *TPP1* transcription in HepG2 and Huh7 cells (Figure 3F,G). These results demonstrated that cisplatin enhanced lysosomal biosynthesis by activating TFEB in HCC, causing synergistic mitochondrial‐lysosomal crosstalk and enhancing mitophagy.

### 3.4 Mitochondrial‐lysosomal crosstalk was important for the resistance of HCC cells to cisplatin

**Figure 4:**
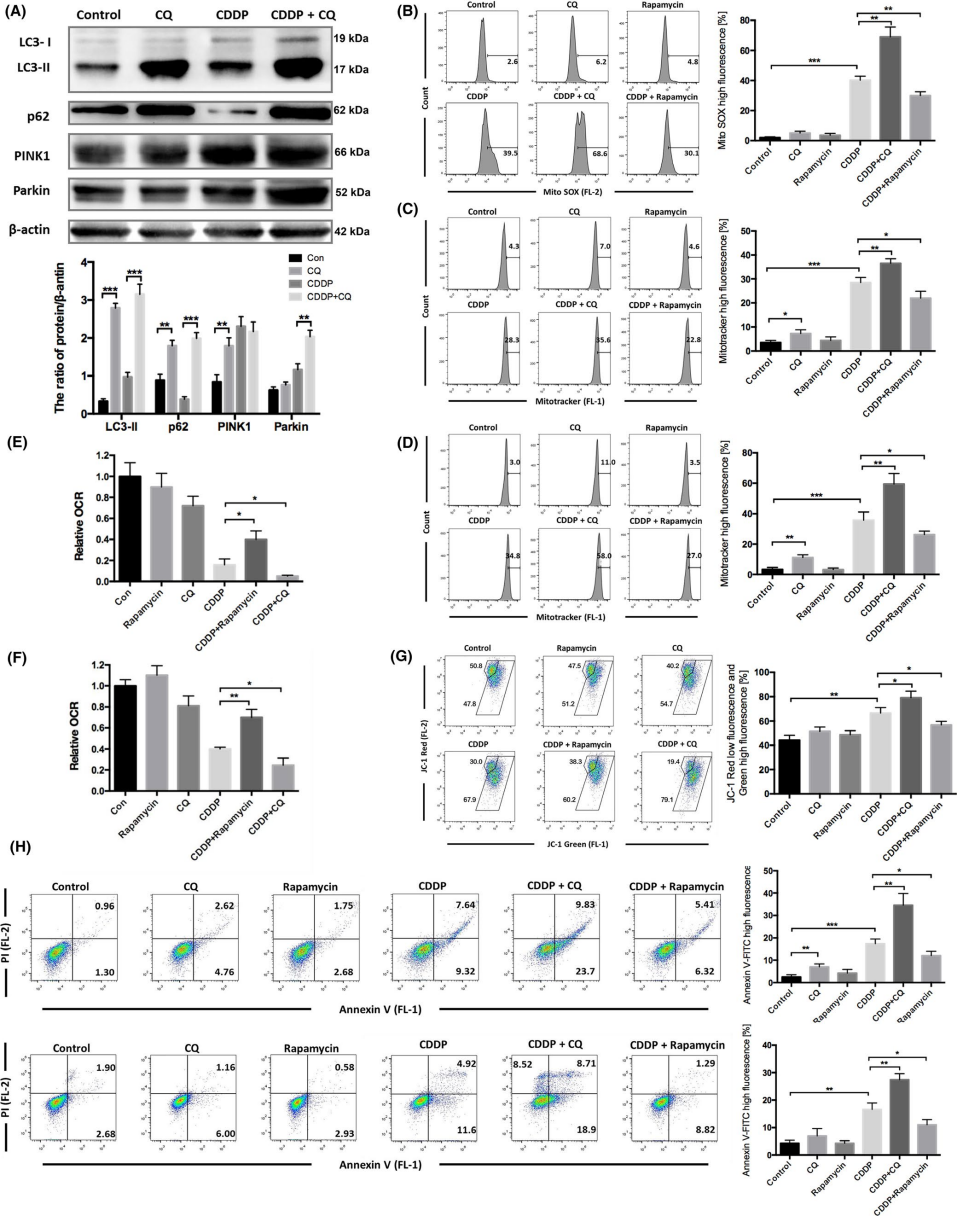
Mitochondrial‐lysosomal crosstalk was important for the resistance of HCC cells to cisplatin. A, Western blot detection of mitophagy‐lysosomal pathway‐related proteins in Huh7 cells treated with 8 μg/mL cisplatin and/or 20 μmol/L CQ for 24 h. The protein/beta‐actin ratio is expressed as the mean ± SD; n = 3, **P < 0.01, ***P < 0.001. B, Huh7 cells were treated with 8 μg/mL cisplatin combined with 20 μmol/L CQ or 5 μmol/L rapamycin for 24 h and then stained with MitoSOX Red and detected using flow cytometry. The percentage of cells with high MitoSOX fluorescence is expressed as the mean ± SD; n = 3, **P < 0.01, ***P < 0.001. C, Huh7 cells (same treatment as A) and D, HepG2 cells were treated with 12 μg/mL cisplatin combined with 20 μmol/L CQ or 5 μmol/L rapamycin for 24 h and then stained with MitoTracker Green FM and detected using flow cytometry. The percentage of cells with high MitoTracker fluorescence is expressed as the mean ± SD; n = 3, *P < 0.05, **P < 0.01, ***P < 0.001. E, OCR was measured after Huh7 cells were treated with 8 μg/mL cisplatin combined with 20 μmol/L CQ or 5 μmol/L rapamycin. Relative OCR values are expressed as the mean ± SD; n = 3, *P < 0.05. F, OCR was measured after HepG2 cells were treated with 12 μg/mL cisplatin combined with 20 μmol/L CQ or 5 μmol/L rapamycin. Relative OCR values are expressed as the mean ± SD; n = 3, *P < 0.05, **P < 0.01. G, Huh7 cells were stained with JC‐1 and analysed by FlowJo. The percentage of cells with low red fluorescence and high green fluorescence is expressed as the mean ± SD; n = 3, *P < 0.05, **P < 0.01. H, Huh7 cells and I, HepG2 cells were stained with Annexin V and PI and analysed by FlowJo. The percentage of cells with high Annexin V‐FITC fluorescence is expressed as the mean ± SD; n = 3, *P < 0.05, **P < 0.01, ***P < 0.001

Treatment of Huh7 cells with cisplatin and CQ caused accumulation of the mitophagy‐related proteins PINK1, parkin, LC3 and p62 (Figure 4A), effectively blocking mitophagy. Rapamycin, an mTOR inhibitor shown to induce mitophagy,^46^, ^47^, ^48^ was used to verify the protective effect of mitophagy. MitoSOX Red staining revealed that treatment with rapamycin enhanced the clearing of cisplatin‐induced mtROS in Huh7 cells, while CQ aggravated cisplatin‐induced mtROS accumulation (Figure 4B). MitoTracker Green staining (Figure 4C,D) and OCR measurement (Figure 4E,F) showed that rapamycin ameliorated the mitochondrial dysfunction and impaired the mitochondrial accumulation induced by cisplatin in HCC cells. Mitochondrial function was further inhibited, and mitochondrial accumulation was aggravated, in the group treated with CQ and cisplatin. We also evaluated the mitochondrial membrane potential using JC‐1 and obtained similar results (Figure 4G). Annexin V‐FITC(+) staining showed that, compared with cisplatin alone, treatment with rapamycin reduced the apoptosis rate in HepG2 and Huh7 cells, while treatment with CQ enhanced cisplatin‐induced apoptosis in HCC cells (Figure 4H,I). Taken together, these results indicated that mitochondrial‐lysosomal crosstalk plays a protective role in the resistance of HCC cells to cisplatin.

### 3.5 Cisplatin combined with the PI3K/mTOR inhibitor PKI‐402 destroyed mitochondrial‐lysosomal crosstalk and induced LMP in HCC cells

**Figure 5:**
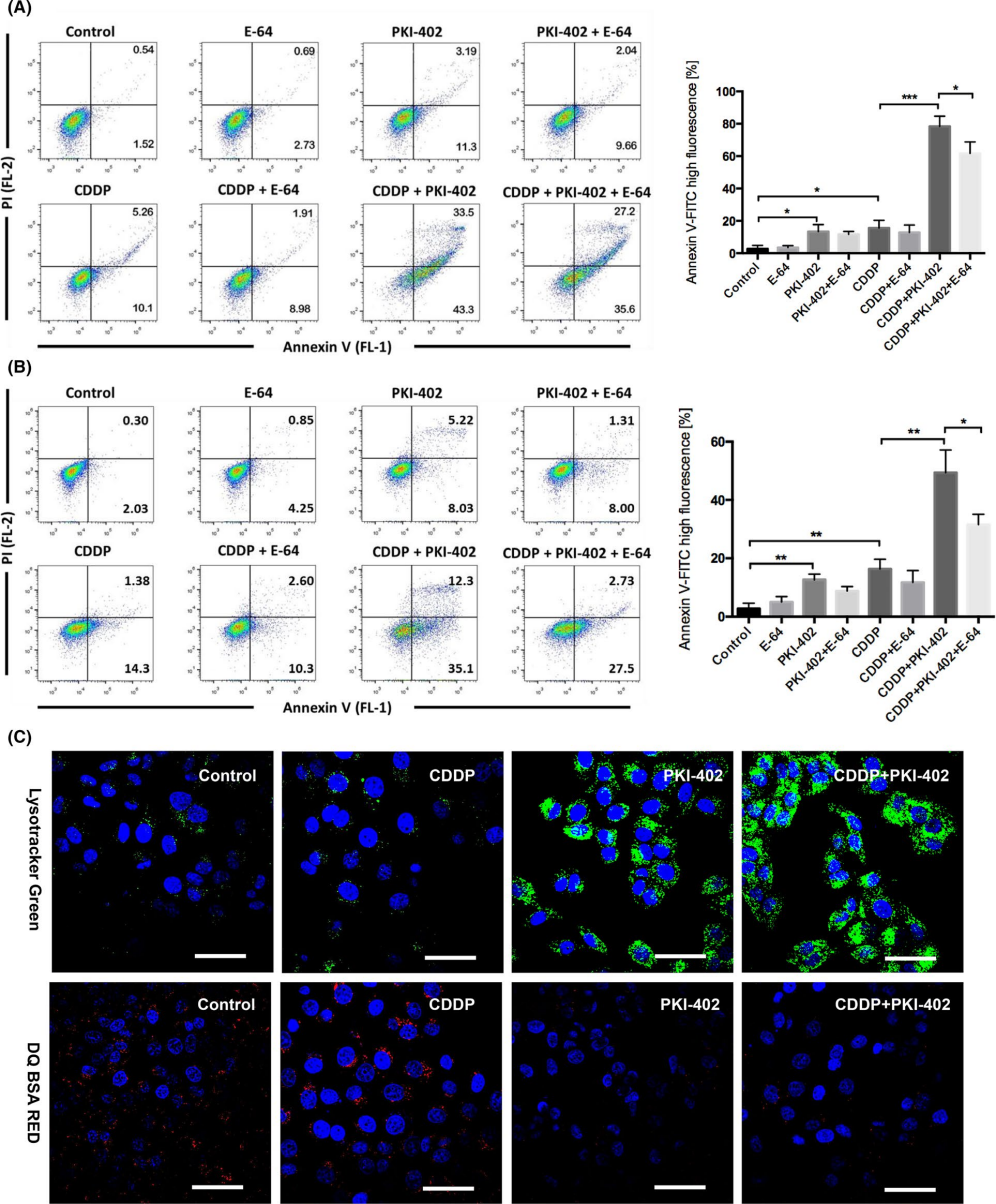
PKI‐402 combined with cisplatin significantly increased apoptosis and induced lysosomal dysfunction in HCC cells. A, Huh7 cells were treated with 8 μg/mL cisplatin and/or 5 μmol/L PKI‐402 in the presence or absence of 5 μmol/L E‐64 for 24 h and then stained with Annexin V and PI and analysed by FlowJo. The percentage of cells with high Annexin V‐FITC fluorescence is expressed as the mean ± SD; n = 3, *P < 0.05, ***P < 0.001. B, HepG2 cells were treated with 12 μg/mL cisplatin and/or 5 μmol/L PKI‐402 in the presence or absence of 5 μmol/L E‐64 for 24 h and then stained with Annexin V and PI and analysed by FlowJo. The percentage of cells with high Annexin V‐FITC fluorescence is expressed as the mean ± SD; n = 3, *P < 0.05, **P < 0.01. C, Huh7 cells were treated with 8 μg/mL cisplatin and/or 5 μmol/L PKI‐402 for 12 h and then stained with LysoTracker Green DND‐26 and DQ Red BSA and observed with confocal laser microscopy (scale bar = 20 μm)

Based on the above results, inhibition of lysosomal function should increase the sensitivity of HCC cells to cisplatin. However, we postulated that if we could alter the protective role of lysosomes to that of a cell death promoter in HCC cells, then mitochondrial‐lysosomal crosstalk would be effectively destroyed and HCC sensitivity to cisplatin would be increased significantly. Annexin V‐FITC(+) staining showed that combined treatment with the PI3K/mTOR inhibitor PKI‐402 and cisplatin induced apoptosis in 76.8% of Huh7 cells and 47.4% of HepG2 cells compared with 15.3% of Huh7 cells and 12.9% of HepG2 cells in the group treated with cisplatin alone, 14.5% of Huh7 cells and 13.3% of HepG2 cells in the group treated with PKI‐402 alone and 2.0% of Huh7 cells and 2.3% of HepG2 cells in the control group (Figure 5A,B). E‐64 can inhibit the activity of many cathepsins, including cathepsin B, reducing the rate of apoptosis induced by PKI‐402 in combination with cisplatin to 62.8% in Huh7 cells and 30.2% in HepG2 cells. We selected the highly sensitive Huh7 cells for further analysis. LysoTracker Green staining revealed a significantly enhanced fluorescence intensity in the group treated with PKI‐402 alone and the group treated with PKI‐402 and cisplatin compared with the group treated with cisplatin alone and the control group (Figure 5C). Interestingly, DQ Red BSA staining showed significantly reduced fluorescence intensity in the group treated with PKI‐402 and cisplatin compared with the control group and the group treated with cisplatin alone, suggesting that PKI‐402 combined with cisplatin damaged lysosomal function.

**Figure 6:**
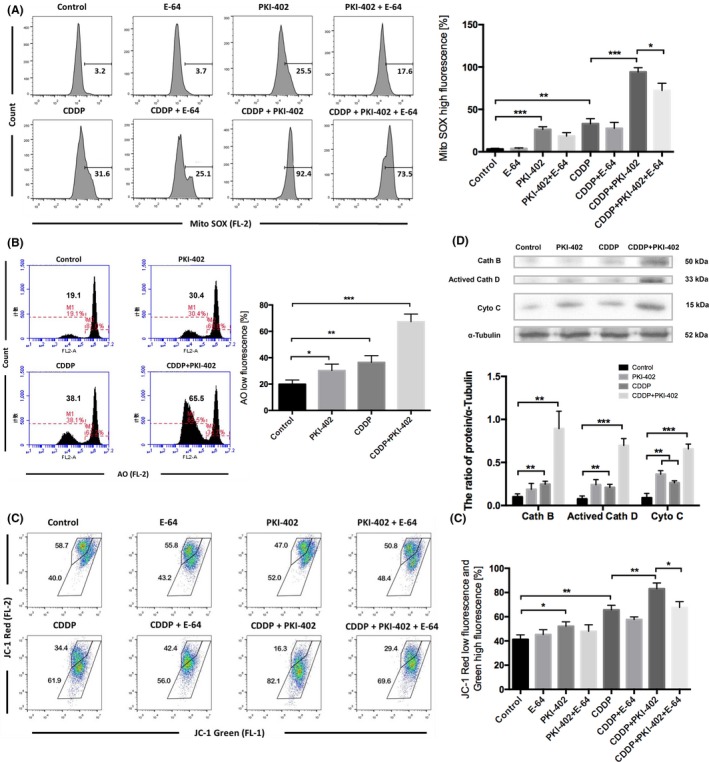
Cisplatin combined with PKI‐402 destroyed mitochondrial‐lysosomal crosstalk and induced LMP and apoptosis. A, Huh7 cells were treated with 8 μg/mL cisplatin and/or 5 μmol/L PKI‐402 in the presence or absence of 5 μmol/L E‐64 for 24 h and then stained with MitoSOX Red and detected using flow cytometry. The percentage of cells with high MitoSOX fluorescence is expressed as the mean ± SD; n = 3, *P < 0.05, **P < 0.01, ***P < 0.001. B, Huh7 cells were treated with 8 μg/mL cisplatin and/or 5 μmol/L PKI‐402 for 12 h and then stained with AO and detected using flow cytometry. The percentage of cells with low AO fluorescence is expressed as the mean ± SD; n = 3, *P < 0.05, **P < 0.01, ***P < 0.001. C, Huh7 cells (same treatment as A) were stained with JC‐1 and analysed by FlowJo. The percentage of cells with low red fluorescence and high green fluorescence is expressed as the mean ± SD; n = 3, *P < 0.05, **P < 0.01. D, Cytoplasmic proteins were extracted, and the levels of cathepsin B, cathepsin D and cytochrome C were detected by Western blotting. The protein/alpha‐tubulin ratio is expressed as the mean ± SD; n = 3, **P < 0.01, ***P < 0.001

PKI‐402 increased the mtROS levels in HCC cells, and the levels were significantly increased by combinatorial treatment with PKI‐402 and cisplatin. Conversely, E‐64 reduced the mtROS levels (Figure 6A). To further confirm that PKI‐402 combined with cisplatin induces LMP in HCC cells, we used acridine orange (AO), a lysosomotropic dye that accumulates in lysosomes and produces red fluorescence. LMP would result in leakage of AO into the cytosol, leading to a decrease in red fluorescence.^39^, ^49^ The red fluorescence intensity of the groups treated with PKI‐402 alone or cisplatin alone decreased slightly, while that of the group treated with PKI‐402 and cisplatin decreased significantly, in Huh7 cells (Figure 6B). Then, we extracted the cytoplasmic proteins and measured the protein levels of cathepsin B, cathepsin D and cytochrome C by Western blotting (Figure 6D). The protein levels in the cytoplasm of cells treated with cisplatin alone or PKI‐402 alone were slightly higher than those of the control group, but the levels were significantly higher than those of the group treated with PKI‐402 and cisplatin. Additionally, JC‐1 staining revealed a significant decrease in the mitochondrial membrane potential of cells treated with PKI‐402 and cisplatin, while E‐64 increased the mitochondrial membrane potential to some extent (Figure 6C). The above results showed that the mtROS levels in HCC cells increased after PI3K/mTOR inhibition and that combinatorial treatment with cisplatin could further increase the mtROS levels in HCC cells, leading to LMP, mitochondrial depolarization, mitochondrial outer membrane permeabilization (MOMP) and apoptosis.

## 4 DISCUSSION

Mitochondria are responsible for regulating various forms of cell death, including apoptosis and necrosis.^50^ Therefore, to resist chemotherapeutic drugs, cancer cells must maintain mitochondrial homeostasis. Although most previous studies have suggested that the main target of cisplatin is nuclear DNA, recent studies have found that mitochondria are critical targets of cisplatin,^11^ and this finding was supported by our present findings in HCC cells. The underlying mechanism may be associated with the genotoxicity of cisplatin towards mtDNA^51^; cisplatin binds to mtDNA with higher efficiency than to nuclear DNA,^52^ but the efficiency of DNA repair in mitochondria is consistently low. However, cells initiate mitophagy to maintain organelle homeostasis. Mitochondrial fission is considered to be a sorting mechanism for mitophagy, as demonstrated by PINK1/parkin‐mediated mitophagy. Loss of the mitochondrial membrane potential will stabilize PINK1 at the outer membrane, which can recruit parkin, leading to mitochondrial fragmentation and subsequent mitophagy.^23^, ^43^, ^53^ However, it is unknown whether there exists a similar mechanism of cisplatin resistance in HCC cells. In the present study, we showed that cisplatin can induce mitochondrial fission and depolarization in HCC cells, which is a precondition of mitophagy.^43^ Western blot analysis showed that cisplatin activated PINK1/parkin‐mediated mitophagy in HCC cells, which selectively targeted impaired mitochondria for degradation. The autophagy inhibitor CQ can inhibit mitophagy effectively and increase the cell death rate, which demonstrates that mitophagy plays a protective role in HCC cells. Zhou et al^54^ found that mitophagy serves a prosurvival function in doxorubicin‐induced breast cancer cell death, which further suggests that mitophagy may be an important mechanism for resistance to chemotherapeutic drugs and maintenance of mitochondrial homeostasis and survival in cancer cells.

Mitophagy involves the constant consumption of a large number of lysosomes. Therefore, we focused on a key transcription factor, TFEB, which links mitophagy and lysosomal biogenesis. On the one hand, TFEB can initiate lysosomal biogenesis^27^ and provide a large number of lysosomes for mitophagy. On the other hand, TFEB promotes the formation of autophagosomes and fusion of autophagosomes with lysosomes, increasing mitophagic flux.^27^ Therefore, TFEB may be an important messenger in mitochondrial‐lysosomal crosstalk. In this study, we found that TFEB mRNA and protein expression are upregulated in HCC cells treated with cisplatin, and cisplatin‐induced TFEB translocated to the nucleus in HCC cells, leading to upregulation of gene expression of the CLEAR network and enhanced lysosomal biogenesis. Most studies have focused on the mechanism of TFEB activation during starvation. However, recent studies have found that mitochondrial stress and ROS production are important mechanisms of TFEB activation.^28^, ^55^ Nezich et al^56^ reported that TFEB translocated to the nucleus during mitophagy and exhibited transcriptional activity in a PINK1‐ and parkin‐dependent manner. Therefore, cisplatin‐induced ROS and mitophagy may activate TFEB in HCC cells. Notably, TFEB can activate PGC‐1α, which is a transcription factor that is responsible for regulation of mitochondrial biogenesis.^57^ Whether PGC‐1α is activated by TFEB to supplement degraded mitochondria by mitophagy and maintain mitochondrial homeostasis needs further verification.

Lysosomal membrane stability is a prerequisite for lysosomal function and mitophagy. PI3K signalling regulates lysosomal maturation, size and activity and is involved in the control of lysosomal stability,^38^, ^39^, ^58^ and PI3K/mTOR inhibitors were reported to cause LMP.^39^, ^59^, ^60^, ^61^ In this study, we showed that cisplatin combined with the dual PI3K/mTOR inhibitor PKI‐402 induced LMP in HCC cells. Recent studies have suggested that mtROS production is a major inducer of LMP.^30^ We also found that PKI‐402 elevated mtROS levels in HCC cells, which were further enhanced by cisplatin. Interestingly, we found that cisplatin caused a low degree of LMP in HCC cells. This finding is consistent with other studies that reported that an interaction between cisplatin and lysosomes caused LMP accompanied by apoptosis, which may be associated with the accumulation of cisplatin in lysosomes, where this compound initiates LMP.

We showed that LMP induced by PKI‐402 combined with cisplatin was the main cause of HCC cell death. Cathepsin B and cathepsin D can perform degradation even in the cytoplasm,^62^, ^63^ and the cathepsin B‐specific inhibitor E‐64 reduced the cell death rate of PKI‐402 in combination with cisplatin, indicating that translocation of cathepsin to the cytosol leads to indiscriminate degradation of cellular components and cell death. LMP has previously been reported to cause apoptotic or necrosis‐like cell death and may be associated with LMP levels. Massive LMP often induces cell death via necrosis, and partial and selective LMP induces cell death via apoptosis.^58^ We further examined the type of cell death induced by LMP in HCC cells. We showed that cisplatin combined with PKI‐402 significantly induced mitochondrial depolarization by JC‐1 staining in HCC cells, and cisplatin combined with PKI‐402 also increased cytoplasmic cytochrome C levels, which suggested the occurrence of MOMP. The results suggested that LMP induces cell death by apoptosis in HCC cells. PKI‐402 combined with cisplatin significantly increased the apoptosis rate of HCC cells compared to CQ combined with cisplatin. This finding suggested that LMP can destroy the mitochondrial‐lysosomal crosstalk of HCC cells, which is more effective than the inhibition of mitochondrial‐lysosomal crosstalk exhibited by CQ. Furthermore, PKI‐402 combined with cisplatin significantly increased the number of lysosomes, which may be associated with the inhibition of mTOR by PKI‐402 and the alleviation of the inhibition of TFEB^64^; this effect increases the quantity of lysosomes in HCC cells and enhances the killing effect of LMP.

We also found that mtROS levels were positively correlated with LMP, mitochondrial depolarization and apoptosis, suggesting that mtROS levels are associated with mitochondrial and lysosomal damage, which further aggravate the accumulation of mtROS. Therefore, mtROS is an important positive feedback regulator that destroys mitochondrial‐lysosomal crosstalk.

**Figure 7:**
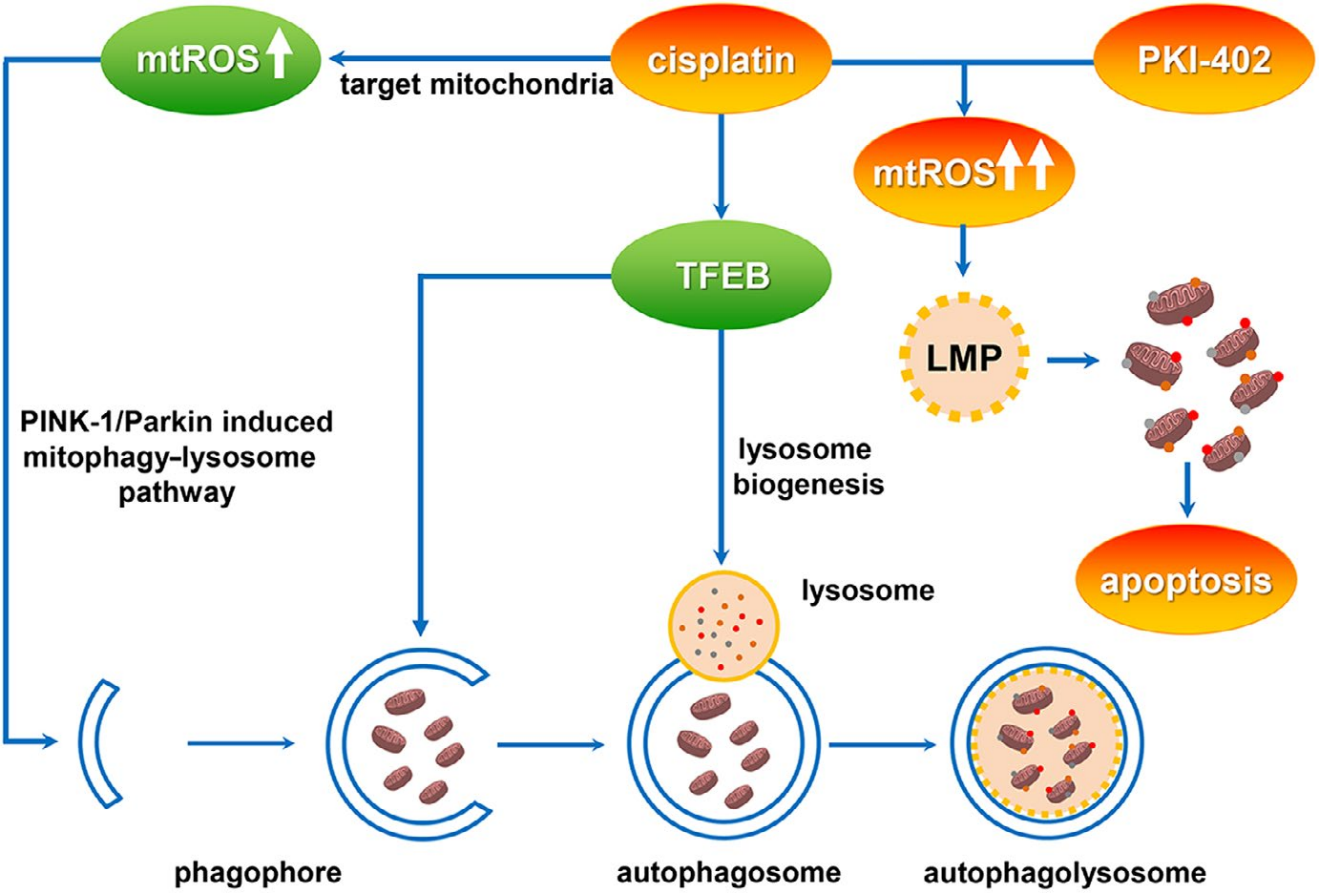
Cisplatin combined with PKI‐402 changed the role of the lysosome from a protective one to that of a cell death promoter in HCC cells

In summary, we provide evidence that mitochondria are important targets of cisplatin in HCC cells. Cisplatin‐induced mitophagy and lysosomal biogenesis constitute mitochondrial‐lysosomal crosstalk, which is a crucial mechanism by which HCC cells overcome the cytotoxicity of cisplatin. We also showed that a combination of cisplatin and the PI3K/mTOR inhibitor PKI‐402 induced LMP in HCC cells and then destroyed cisplatin‐induced mitochondrial‐lysosomal crosstalk, which significantly increased the sensitivity of HCC cells to cisplatin (Figure 7). This study will provide new ideas and candidate targets for comprehensive treatment of HCC.

## CONFLICT OF INTEREST

The authors declare that they have no conflict of interest.
