# Supplementary material for: Inhibition of PI3K/mTOR increased the sensitivity of hepatocellular carcinoma cells to cisplatin via interference with mitochondrial‐lysosomal crosstalk
Source: Cell Prolif. 2019 Apr 29;52(3):e12609. doi: 10.1111/cpr.12609 (PMC6536453; doi:10.1111/cpr.12609)
Supplement: Supplementary file 1 [file CPR-52-e12609-s001.docx]

| Layout | 1 | 2 | 3 | 4 | 5 | 6 | 7 | 8 | 9 | 10 | 11 | 12 |
| --- | --- | --- | --- | --- | --- | --- | --- | --- | --- | --- | --- | --- |
| **A** | ATP12A | ATP4A | ATP4B | ATP5A1 | ATP5B | ATP5C1 | ATP5F1 | ATP5G1 | ATP5G2 | ATP5G3 | ATP5H | ATP5I |
|  | 227.76 | 792.86 | 119.92 | ­5428.51 | ­947.18 | ­61.38 | ­4.34 | ­4.29 | 527.67 | ­281.26 | ­69.76 | ­5483.73 |
| **B** | ATP5J | ATP5J2 | ATP5L | ATP5O | ATP6V0A2 | ATP6V0D2 | ATP6V1C2 | ATP6V1E2 | ATP6V1G3 | BCS1L | COX4I1 | COX4I2 |
|  | ­16.25 | 22.33 | 23.88 | ­29.25 | 10.61 | 20892.46 | 7315.13 | 4.87 | 365.55 | 4.93 | ­18.83 | 345.29 |
| **C** | COX5A | COX5B | COX6A1 | COX6A2 | COX6B1 | COX6B2 | COX6C | COX7A2 | COX7A2L | COX7B | COX8A | COX8C |
|  | ­39.41 | ­7.58 | ­20.83 | 720.03 | ­1175.74 | 480.82 | 2406 | ­26.29 | ­9.30 | 1.6 | ­16.01 | 1874.2 |
| **D** | CYC1 | LHPP | NDUFA1 | NDUFA10 | NDUFA11 | NDUFA2 | NDUFA3 | NDUFA4 | NDUFA5 | NDUFA6 | NDUFA7 | NDUFA8 |
|  | ­53.69 | ­3.13 | 1.28 | ­2.32 | ­16.45 | ­24.03 | ­1.99 | ­3.48 | ­11.25 | ­2.57 | ­26.11 | ­11.70 |
| **E** | NDUFAB1 | NDUFB10 | NDUFB2 | NDUFB3 | NDUFB4 | NDUFB5 | NDUFB6 | NDUFB7 | NDUFB8 | NDUFB9 | NDUFC1 | NDUFC2 |
|  | ­50.41 | ­3.94 | ­14.30 | ­76.01 | 1.42 | ­29.82 | ­289.98 | ­19.53 | ­14.57 | 2.4 | 3.22 | 41.48 |
| **F** | NDUFS1 | NDUFS2 | NDUFS3 | NDUFS4 | NDUFS5 | NDUFS6 | NDUFS7 | NDUFS8 | NDUFV1 | NDUFV2 | NDUFV3 | OXA1L |
|  | ­11.45 | ­14.33 | 1.14 | 2.88 | ­13.90 | ­38.36 | ­1.08 | ­14.89 | ­1.43 | ­2.43 | 3.07 | ­27.41 |
| **G** | PPA1 | PPA2 | SDHA | SDHB | SDHC | SDHD | UQCR11 | UQCRC1 | UQCRC2 | UQCRFS1 | UQCRH | UQCRQ |
|  | ­1.17 | 1.87 | ­4.40 | ­6.42 | ­17.93 | 3.38 | 8.47 | 1.04 | ­802.44 | ­20.0 | ­6377.21 | 1.14 |

Supplementary Table 1.
